# A Universal Base in a Specific Role: Tuning up a Thrombin Aptamer with 5-Nitroindole

**DOI:** 10.1038/srep16337

**Published:** 2015-11-17

**Authors:** Vladimir B. Tsvetkov, Anna M. Varizhuk, Galina E. Pozmogova, Igor P. Smirnov, Natalia A. Kolganova, Edward N. Timofeev

**Affiliations:** 1 W. A. Engelhardt Institute of Molecular Biology Russian Academy of Sciences, Moscow 119991, Russia; 2 Institute for Physical-Chemical Medicine, Moscow 119435, Russia; 3 Topchiev Institute of Petrochemical Synthesis Russian Academy of Sciences, Moscow 119991, Russia

## Abstract

In this study we describe new modified analogs of the thrombin binding aptamer (TBA) containing 5-nitroindole residues. It has been shown that all modified TBAs form an anti-parallel G-quadruplex structure and retain the ability to inhibit thrombin. The most advanced TBA variant (TBA-N8) has a substantially increased clotting time and two-fold lower IC_50_ value compared to the unmodified prototype. Molecular modelling studies suggest that the improved anticoagulant properties of TBA-N8 result from changes in the binding mode of the analog. A modified central loop in TBA-N8 is presumed to participate in the binding of the target protein. Studies of FAM labelled TBA and TBA-N8 showed an improved binding affinity of the modified aptamer and provided evidence of a direct interaction between the modified central loop and thrombin. Our findings have implications for the design of new aptamers with improved binding affinities.

A thrombin binding aptamer (TBA) is a synthetic 15-nt DNA oligomer that binds human alpha thrombin with high specificity and reversibly suspends the coagulation cascade and platelet formation. It has been established that TBA folds intramolecularly into a G-quadruplex (GQ) structure in solution, forming two G tetrads and three loops^1^. Two TT loops play a critical role in recognizing the target protein at its fibrinogen binding site (exosite I). The most recent high-resolution crystallographic studies of the TBA-thrombin complex have provided details of the interaction between thrombin and TBA loops^2^,^3^. It was shown that, in TT loops, T4 and T13 form polar interactions with the amino acid residues of exosite I, while T3 and T12 form hydrophobic contacts. The role of the central TGT loop has been arguably attributed to the binding of the aptamer to the heparin binding site of thrombin (exosite II)^4^,^5^,^6^. However, a recent crystallographic study suggests that the TGT loop is unlikely to be involved in thrombin binding and interacts with neither the fibrinogen nor the heparin binding sites of the protein^3^. Although located away from the binding site of the aptamer, the central loop is a key element of TBA structure. Numerous studies on modifications of TBA with non-natural nucleotides indicate that the improved anticoagulant properties or higher binding affinities of modified variants are often associated with the transformation of the TGT loop. In this way, a few advanced TBA analogs containing 2′-deoxyisoguanosine, 2′-deoxy-2′-fluoroarabinonucleotides, 4-thio-2′-deoxyuridine, 5-hydroxymethyl-2′-deoxyuridine, D-/L-isothymidine and UNA (unlocked nucleic acid) residues in the TGT loop have been described^7^,^8^,^9^,^10^,^11^,^12^.

Artificial residues that have been used for numerous TBA modifications feature altered sugar units, nucleobases, and an internucleotide backbone^13^. Hydrophobic interactions in TBA studies have mainly been considered in the context of binding intercalators and fluorophores^14^,^15^,^16^,^17^,^18^. Replacement of G nucleobases within TBA G-tetrads with fluorescent 8-aryl-dG residues has recently been reported^19^. TBA analogs containing universal bases have not been described. 5-Nitroindole (NI) has long been known as the best universal base analogue for double stranded DNA^20^,^21^. This non-natural nucleoside has been shown to interact with DNA base pairs *via* π−π stacking^22^ and may be a good candidate to improve the biophysical and/or biochemical properties of TBA when added to the TGT loop region.

In this study, we describe, for the first time, the modification of TBA with NI residues. Modified oligonucleotides were designed to place a non-natural NI unit distant from the TT loops comprising the aptamer recognition site. We found that four of the five modified variants exhibit specific anticoagulant activity very close to that of TBA regardless of the location of the NI residue and thermodynamic stability of the GQ structure. Unexpectedly, a considerable increase of antithrombotic ability was observed for a single TBA variant, TBA-N8. Detailed studies of this aptamer evidence an important role of the central T(NI)T loop in binding thrombin.

## Results and Discussion

### Anticoagulant activity of modified aptamers

**Figure 1:**
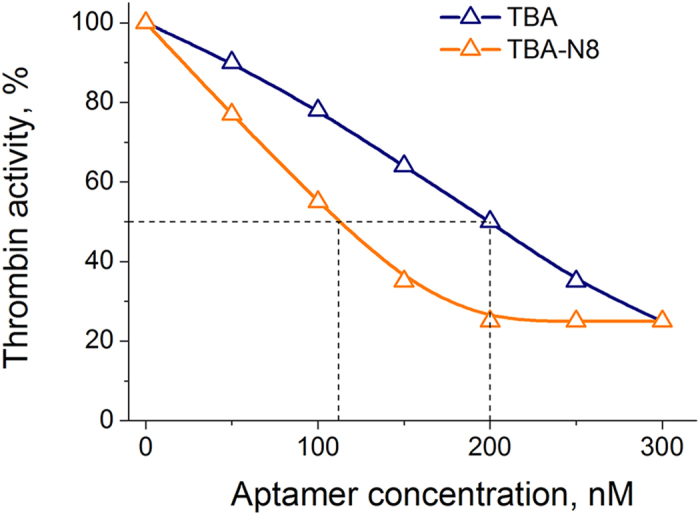
Thrombin activity *vs*. aptamer concentration. The clotting times measured in the presence of the aptamers were converted into thrombin activity values using a calibration curve (Supplementary Figure S14).

We synthesized five modified TBA analogs containing either NI substitutions in the TGT loops or additional NI nucleotides at the 3′ or 5′ end (Table 1). Clotting time tests assessed the inhibitory properties of the modified aptamers. All modified TBA analogs retained specific anticoagulant activity, as shown in Table 1. Moreover, oligonucleotide N8 was characterized by a substantially increased clotting time compared to TBA. The specific effect of the G8 substitution on the inhibitory activity of TBA is shown in Fig. 1. An IC_50_ value for TBA-N8 was half that for TBA. In aptamers TBA-N7, TBA-N9, TBA-N1e and TBA-N15e, the minimum effect of NI modifications on the biological properties of the TBA analogs supports the modern vision of the aptamer binding pattern via the TT loops. Indeed, the antithrombotic activity was not altered in the four TBA variants containing NI residues away from the binding site. In contrast, the modified aptamer TBA-N8 demonstrated a considerable enhancement of antithrombotic properties. Such a difference may generally imply a notable increase in the nuclease resistance of TBA-N8, as well as significant changes in the structure or binding mode of this analog. To verify all of these possibilities, we examined the stability of the aptamers in an S1 nuclease cleavage assay. Additionally, we characterized the GQ structure of the aptamers by spectroscopic methods, and compared TBA and TBA-N8 in molecular modelling studies.

### The anticoagulant activity of aptamers does not correlate with their nuclease resistance

A modification to the loop nucleotides in TBA may result in significantly higher nuclease resistance for the modified aptamer. For example, extraordinary nuclease resistance has been reported for anomeric modification of TBA in loop regions^23^. An increase in nuclease resistance may potentially affect the results of clotting time tests due to slower degradation of the modified TBA variants in blood plasma. Cleavage of TBA and NI variants with S1 nuclease produced very similar patterns of degradation (Supplementary Figure S1) with insignificant variations. Unmodified TBA appears to be somewhat more stable than the NI analogs. The lowest stability was observed for aptamers TBA-N7 and NBA-N1e. Evidently, in this case differences in nuclease resistance cannot account for the observed enhancement of the anticoagulant activity of TBA-N8.

### Spectroscopic characterization of intramolecular G-quadruplexes

The formation of GQ structures by all of the modified oligonucleotides was supported by observation of UV melting at 295 nm. The intramolecular folding mode was confirmed by the fact that all aptamers did not show any significant hysteresis at the temperature ramp rate of 0.5 °C/min (Supplementary Figure S2). Thermal denaturation studies of TBA variants in 100 mM KCl produced a notable destabilizing effect for the T substitutions in TBA-N7 and TBA-N9. The decrease in T_m_ for TBA-N7 and TBA-N9 was 9 and 13 °C, respectively. Interestingly, despite the low thermodynamic stability, TBA-N7 and TBA-N9 are still able to inhibit thrombin. Based on their T_m_ values, approximately 50% of either aptamer is expected to be unfolded at 37 °C. The only feasible explanation of such an effect may consist in the stabilizing role of thrombin as a molecular chaperon. The mechanism of action is based on interactions between the protein and certain aptamer nucleobases and does not involve G-quartets^24^.

The T_m_ values for G8-substituted TBA-N8, as well as for analogs TBA-N1e and TBA-N15e, are close to the T_m_ value of the unmodified TBA. In TBA-N8, the universal base seems to be a good equivalent of guanine G8, which is stacked to the nearest G-quartet in TBA^1^. This is not the case for TBA-N9, given that in TBA, T9 is stacked with the same G-quartet. NI caps at the 3′ and 5′ ends did not add to aptamer stability. In duplex DNA, the stabilizing effect of an additional NI base has been well studied^20^,^21^. What is most likely is that the lack of stabilization for TBA-N1e and TBA-N15e is due to the missing interaction between an extra base and the GQ core.

**Figure 2:**
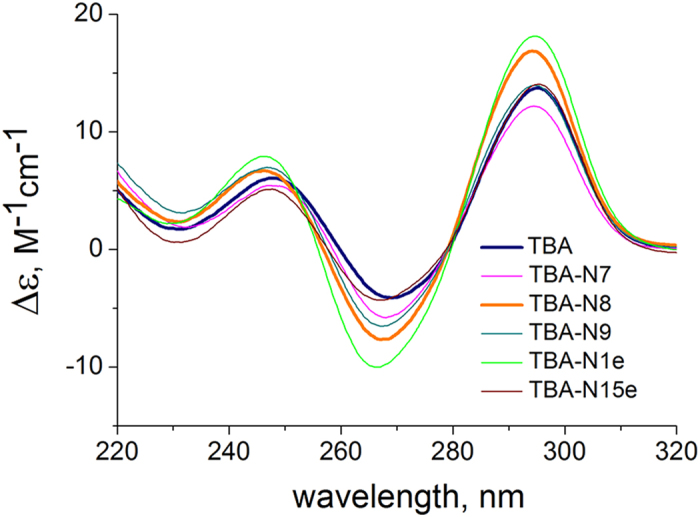
CD spectra of TBA and modified aptamers at 20 °C and an aptamer concentration of 5 μM in 10 mM sodium cacodylate (pH 7.2) and 100 mM KCl.

The thermal difference spectra (TDS) of the modified aptamers show a highly specific TBA signature for all the modified variants (Supplementary Figure S3), with two positive peaks at 240 and 275 nm and two negative peaks at 260 and 295 nm. These observations suggest minor or no changes in TBA structure upon modification with NI. Circular dichroism (CD) spectra (Fig. 2) also confirm that all modified aptamers are structurally analogous to TBA. Two positive bands at 245 nm and 295 nm and a negative band at 265 nm support the anti-parallel folding geometry. Thermal denaturation profiles monitored by CD band at 295 nm reproduced UV melting data for all aptamers (Supplementary Figure S4). Thus, the increased activity of TBA-N8 cannot be attributed to substantial refolding and is likely due to a different binding mode.

### Molecular modelling studies

**Figure 3:**
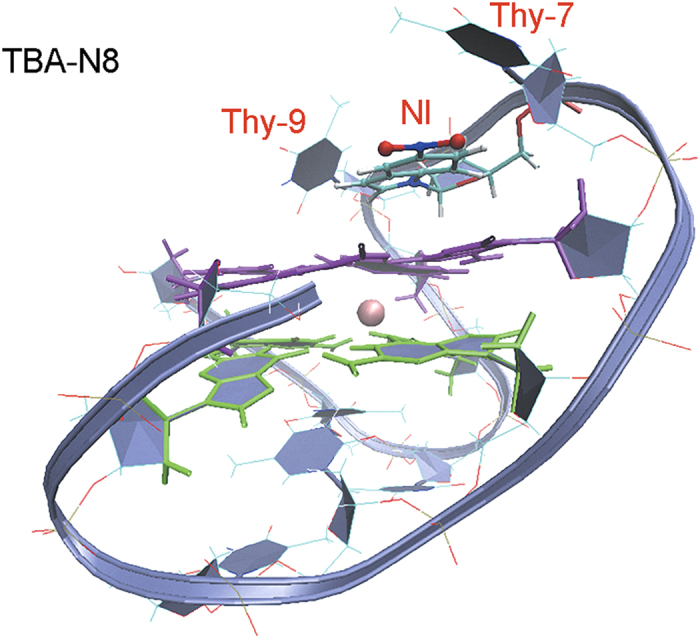
MD simulation results. A 50 ns snapshot of TBA-N8.

We used molecular modelling experiments to clarify the conformations of TBA analogs and elucidate the possible patterns of their interaction with thrombin. The initial models for all modified aptamers were built using unmodified TBA (PDB ID 148D). In TBA-N8, guanine in the central loop was substituted for NI and the structure was reoptimized. Analogous procedures were applied to other aptamers: the respective nucleobases were substituted with NI (TBA-N7 and TBA-N9) or the NI-bearing nucleotide residue was added at 5′/3′- TBA terminus (TBA-N1e and TBA-N15e, respectively). A 50 ns molecular dynamics (MD) simulation was performed. The profiles of root mean square deviations (RMSD) (Supplementary Figure S5) confirm that the final conformations are relatively stable. In all TBA analogs except for TBA-N7, the NI residue tends to participate in stacking with core guanines or loop bases. In TBA-N7, it is brought into close proximity with Gua-5. In TBA-N1e, TBA-N9, and TBA-15e, it is stacked with Gua-8, Gua-15 and Thy-9, respectively (Supplementary Figures S6 and S7). As evident from Fig. 3, NI in TBA-N8 is loosely stacked with the neighboring G tetrad, while Thy-7 and Thy-9 “embrace” it, forming a housetop-like structure. The dynamics of the central loop residues in TBA-N8 suggest a symmetrical structure with equal angles between the planes and equal COM (centre of mass) distances for the pairs NI–Thy7 and NI—Thy-9 after approximately 30 ns (Supplementary Figure S7). As compared to other aptamers, TBA-N8 is relatively ‘loose’ (non-compact in the central loop region), which is evidenced by a higher value of its potential energy (Supplementary Figure S8A). Van der Waals interactions within the aptamer and the interactions with the solvent (Supplementary Figure S8B–D) contribute most significantly to the difference between the normalized energies of the TBA analogs. Thus, TBA-N8 appears somewhat more prone to conformational changes, which may be advantageous for binding with thrombin.

Importantly, no significant rearrangements in the quadruplex core or the TT loops were observed during the simulation. This supports the hypothesis that the increased inhibitory activity of TBA-N8 is conditioned by factors other than conformational rearrangements in the TT epitopes and that the central loop may also be involved in thrombin binding. We performed docking experiments to verify the latter assumption.

The thrombin model used in the docking experiments was taken from PDB ID 1HAO. TBA-N8 was first docked to the whole protein in a fixed conformation (‘rigid docking’) to determine the major binding sites. Control docking of the unmodified TBA was also performed. Predictably, the major putative binding sites were those containing multiple positively charged amino acid residues (Arg and Lys) and included thrombin exosites I and II (Supplementary Figure S9).

Interactions in exosites I and II were further investigated using a different docking procedure: the main body of the protein was fixed, but the amino acid side groups in exosites I and II were flexible. The quadruplex core of TBA/TBA-N8 was fixed, while the loops were partially flexible (rotation of the heterocyclic bases around the glycosidic bond was allowed).

The results of the simulation suggest that the aptamers are able to recognize both sites of thrombin. Two interaction patterns are possible in each case: binding via the TT-loops of the aptamer and binding via the central loop (Supplementary Figure S10). This result is not surprising in view of previous reports on different modes of interaction between TBA and its analogs with thrombin^4^,^25^. Although TBA is generally believed to target exosite I via TT loops, the exact pattern of TBA binding with thrombin is still a matter of debate. To identify the difference between TBA-N8 and TBA in their binding modes, we estimated and compared the binding energies for all four situations (Table 2). It should be taken into account that a different number of amino acid side chain residues was rendered flexible upon docking to exosites I and II (for more details, see the experimental section), which hampers reliable analysis of relative changes in the protein internal strain for the two situations. Thus, absolute values of energy gains can only be compared directly within one exosite.

As seen from Table 2, aptamer interactions with thrombin exosites result in comparable energy gains. However, the average energy of interaction is higher for the modified aptamer. This additional energy gain for TBA-N8 originates largely from the contribution of desolvation and van der Waals and hydrogen bond interactions and has the most notable values for binding *via* the central loop. The highest difference between TBA and TBA-N8 was observed for the interaction *via* the central loop in exosite I (−4.11 vs. −6.65 kcal/mol). The increased binding energies of the trombine-TBA-N8 complex are mainly conditioned by the hydrophobic interactions of the NI base and hydrogen bonding of the NO_2_ group. In particular, binding of TBA-N8 via the central loop benefits from the enhanced ability of the electronegative nitro group to form hydrogen bonds with Arg75 (the fibrinogen binding site) or Asn179 (the heparin binding site) (Supplementary Figure S11).

To verify whether NI residue plays any significant role in the interactions of other TBA analogs with thrombin, we performed ‘half-rigid’ docking of these analogs to exosites I and II. Estimation of the additional energy gains for less potent NI aptamers clearly evidences the dominance of TBA-N8 in binding with the central loop (Supplementary Table 1). Only TBA-N15e showed higher energy gain (E_vdW_ + E_h-bond_ + E_desolv_) upon binding *via* the central loop in exosite I. However, this contribution is comparable with the energy change upon binding *via* the TT loops, thus making prevalence of altered binding mode insignificant. On the contrary, in TBA-N8, central loop binding mode is preferable in both exosites and conditioned by substantional energy changes.

As for the primary target site, free energy calculations argue in favour of thrombin exosite II. Nevertheless, we have to acknowledge this conclusion with a caution, as our data were obtained without taking in account the changes in the internal strain (as mentioned above) and the loss of conformational mobility of the aptamer (for more details, see the experimental section).

Thus, molecular modelling studies provide evidence regarding the altered binding mode of TBA-N8. An important role of the central loop in binding thrombin gives the most reasonable explanation for the enhanced biological activity of TBA-N8.

### Studies of FAM labelled TBA and TBA-N8

**Figure 4:**
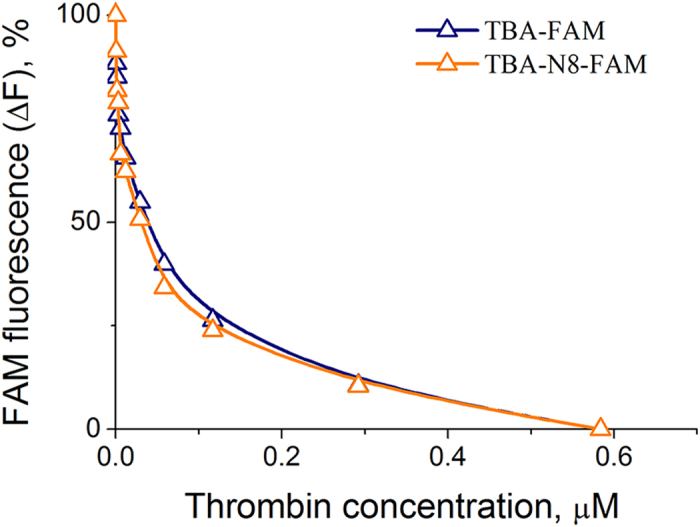
Quenching of FAM fluorescence in labelled aptamers (20 nm) at 20 °C upon binding thrombin in 10 mM sodium cacodylate (pH 7.2) and 100 mM KCl. Fluorescence was calculated as (F–F_min_)/(F_max_–F_min_) × 100%.

To estimate the binding affinity of TBA-N8, we used a fluorescence quenching approach in FAM labelled aptamer upon binding with thrombin. Quenching of a fluorophore by the protein to which it is linked is a known phenomenon and depends on the amino acid environment in the proximity of a dye molecule^26^. This mechanism is different from displacement of fluorophore molecules bound to an aptamer with the protein^15^,^17^. Two oligonucleotide models of TBA-FAM and TBA-N8-FAM were used in our binding experiments. The FAM residue was placed at the 3′-end of either aptamer and spaced from the 3′ dG nucleotide by the hexaethyleneglycol linker. Quenching of FAM fluorescence was confirmed by a decrease of aptamer fluorescence upon binding thrombin. The results of the fluorescence quenching experiments in TBA-FAM and TBA-N8-FAM are shown in Fig. 4. It is clearly shown in Fig. 4 that TBA and TBA-N8 have similar affinities for thrombin. However, the reliability of this conclusion is questionable because of the possible non-specific FAM-protein interactions and/or FAM interference with the specific binding due to its proximity to the central loop of the aptamer. Moreover, FAM fluorescence may only be sensitive to a single binding event, while any further interactions could not be reliably detected.

**Figure 5:**
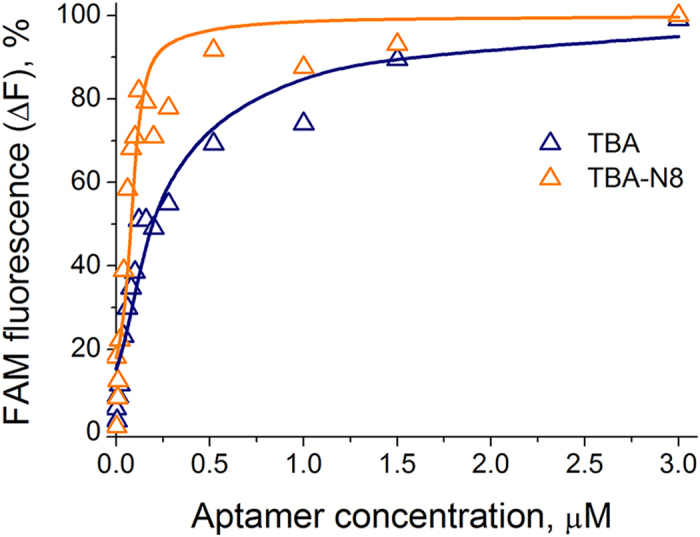
Recovery of fluorescence by TBA and TBA-N8 in displacement experiments. Fluorescent complex thrombin—(TBA-FAM) was formed at a 20 nm aptamer concentration at 20 °C in 10 mM sodium cacodylate (pH 7.2) and 100 mM KCl. Thrombin concentration was 100 nM. Fluorescence was calculated as (F–F_min_)/(F_max_–F_min_) × 100%.

To minimize the possible effect of FAM residue, we changed the scheme of the experiment. Unlabeled TBA and TBA-N8 were used to displace TBA-FAM from its complex with thrombin using five-fold protein excess. The addition of increasing amounts of TBA or TBA-N8 restored FAM fluorescence with notably differing efficiencies as shown in Fig. 5. Experimental data were fitted using a model of competitive binding of two different ligands to a protein molecule^27^ assuming 1:1 stoichiometry. The k_d_ values were 11.3 ± 2.6 nM *vs*. 15.8 ± 5.7 nM for the pair TBA-FAM/TBA, and 18.3 ± 4.9 nM *vs*. 2.3 ± 1.5 nM for the pair TBA-FAM/TBA-N8. When the k_d_ value for TBA-FAM was fixed at 15 nM, an even larger difference between the two unlabeled aptamers was obtained: 23.7 ± 4.8 *vs*. 1.7 ± 0.9 nM. This result has a few important implications: (i) the FAM residue shields in some way the effect of NI in quenching experiments, (ii) loop modification makes TBA-N8 a more potent competitor than TBA, and (iii) both TBA and TBA-N8 bind thrombin exosite I. An increased binding efficiency of unlabeled TBA-N8 and a shielding effect of FAM suggest that the modified central loop may gain an ability to participate in thrombin recognition. Whether this ability simply leads to 2:1 stoichiometry or it is realized in proximity of exosite I is not clear. To elucidate aspects of this binding event we compared TBA-FAM and TBA-N8-FAM in fluorescence polarization studies and native gel-electrophoresis.

**Figure 6:**
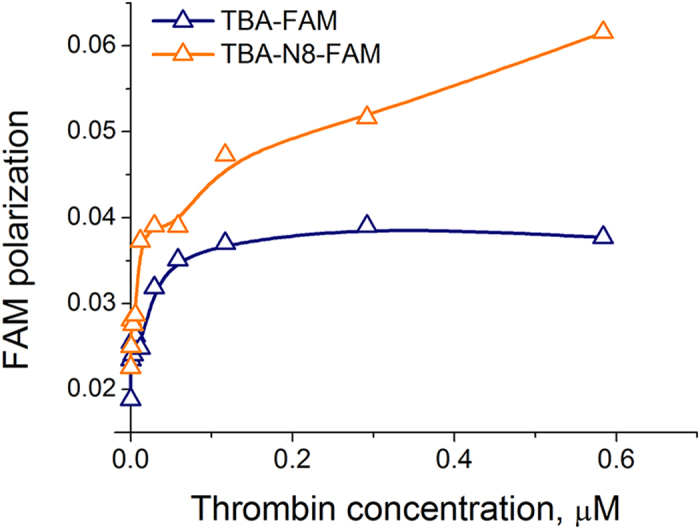
Polarization of FAM fluorescence in labelled aptamers (20 nM) at 20 °C upon binding thrombin in 10 mM sodium cacodylate (pH 7.2) and 100 mM KCl.

Analysis of polarization anisotropy is a fluorescence-based technique for studying large biomolecules using small fluorophore ligands^28^. When exposed to a polarized source, a fluorescent ligand emits light, which is depolarized to the extent the molecule is free to rotate. This method is ideally suited to compare binding of TBA-FAM and TBA-N8-FAM with thrombin if we suspect differences in binding stoichiometry. Despite the above-mentioned shielding effect of the FAM residue, we observed a notable difference between the two aptamers. Representative titration curves are shown in Fig. 6. A biphasic saturation curve for TBA-N8-FAM and a roughly two-fold increase in polarization in the second wave suggests the presence of 2:1 complex in solution at relatively high protein concentrations. The later circumstance clearly identifies the T(NI)T loop as an alternative binding region. Native gel-electrophoresis of the two labelled aptamers in polyacrylamide provides further support for this view (Supplementary Figure S12). An excess of TBA-N8-FAM induces formation of the second slow-migrating band. However, we were not able to observe a 2:1 complex in the gel when an unlabeled modified aptamer was used. Titration of thrombin with TBA-N8 showed only a single protein-aptamer complex. These results repeatedly raise a question about the role of the FAM residue in binding events. At this point, any decisive conclusion on the exact stoichiometry is debatable. Additionally, we cannot identify the loop in TBA-N8 that initiates binding with thrombin. Nevertheless, it is clear that the modified central loop is recruited in binding TBA-N8 with thrombin.

In summary, we have demonstrated that the modification of TBA with NI away from the TT loop region does not impair the anticoagulant activity of the aptamer. We show that all modified TBA variants fold intramolecularly, forming anti-parallel GQ structures. The substitution of G8 for NI in TBA-N8 led to substantial improvement in the anticoagulant properties and binding affinity of the aptamer. Molecular modelling suggests that this effect is due to the changes in the binding mode of TBA-N8, which gains an ability to interact with thrombin *via* the central loop. Studies of FAM labelled aptamers evidence the higher binding affinity of TBA-N8 and provide evidence of the direct interaction of the modified central loop with thrombin.

## Methods

### Oligonucleotides

DNA oligomers were synthesized using an ABI 3400 DNA/RNA synthesizer and purified by reverse phase HPLC. Nitroindole phosphoramidite was purchased from Glen Research, Sterling, VA. Oligonucleotide composition was confirmed by MALDI mass spectrometry using a Bruker Reflex IV mass spectrometer with hydroxypicolinic acid or 2-amino-5-nitropyridine as a matrix.

### UV thermal denaturation and thermal difference spectra

Absorbance *vs*. temperature profiles were obtained with a Chirascan CD spectrometer (Applied Photophysics) equipped with a Peltier cell holder. Melting experiments were carried out at 295 nm in 10 mM sodium cacodylate (pH 7.2) and 100 mM KCl. The heating/cooling rate was 0.5 °C/min. Melting points were determined from derivative plots of the melting curves. Thermal difference spectra were calculated from UV spectra of aptamers at 80 and 20 °C taken with a Shimadzu UV160A spectrophotometer. The oligonucleotide concentration was in the range of 2–6 μM.

### CD spectroscopy

Circular dichroism measurements were performed at 20 °C and an aptamer concentration of 5 μM in 10 mM sodium cacodylate (pH 7.2) and 100 mM KCl with a Jasco-715 CD spectrometer equipped with a Peltier temperature controller. Three scans were performed on a spectra at a bandwidth of 1 nm and a spectral resolution of 0.2 nm. CD melting experiments were carried out with a Chirascan CD spectrometer (Applied Photophysics) at 295 nm.

### Fluorescence quenching and displacement experiments

Fluorescence measurements were carried out in 10 mM sodium cacodylate (pH 7.2) and 100 mM KCl at 20 °C with a Cary Eclipse fluorescent spectrophotometer (Agilent Technologies) equipped with a Peltier cell holder. Excitation and emission wavelengths were 490 and 520 nM, respectively. The concentration of the FAM labelled aptamer was 20 nM. The thrombin concentration varied in the range of 0–580 nM in the quenching experiments. In displacement assays, the thrombin concentration was 100 nM, while the unlabeled aptamer concentration varied in the range of 0–3 μM. To establish equilibrium, the solution was kept at 20 °C for 5 min before each measurement. A minimum of three measurements were made for every mixture. Dilution of the sample was taken into account.

### Fluorescence polarization assay

Thrombin (Sigma) in 10 mM sodium cacodylate (pH 7.2) and 100 mM KCl was added to the solution of the FAM labelled aptamer (20 nM) in the same buffer, and the mixture was kept at 20 °C for 5 min prior to fluorescence measurements. The fluorescence polarization (P) was calculated according to the formula:


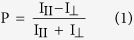


Vertical (I_II_) and horizontal (I_⊥_) components of FAM fluorescence were measured with a Cary Eclipse fluorescence spectrophotometer (Agilent Technologies) upon excitation by vertically polarized light. The excitation wavelength was 490 nm, and the fluorescence intensity was registered at 520 nm.

### Native gel-electrophoreses

All aptamers were annealed in 1xTBE buffer (pH 8.3) containing 100 mM KCl. FAM-labelled aptamers (2 μM) were incubated with increasing concentrations of thrombin for 20 min at 20 °C in a total volume of 10 mL. Additionally, thrombin (4 μM) was incubated with varying concentrations of unlabeled aptamer for 20 min at 20 °C. The bands were resolved in a non-denaturing 10% (for unlabeled aptamers) or 12% (for FAM-aptamers) polyacrylamide gel (19:1) in 1xTBE buffer containing 10 mM KCl. Fluorescent complexes were visualized using a research custom-made imaging system. Unlabelled aptamer-protein complexes and thrombin were visualized with coomassi blue R250.

### Clotting time measurements

Clotting time was measured according to a published procedure^29^ and the manufacturer’s protocol (“Thrombin-TEST”, Renam, Russia). The thrombin-aptamer complex was prepared as follows: 5 μL of the preannealed 20 × (0.5–3 μM) aptamer solution in 20 mM Tris-HCl buffer (pH 7.6) containing 100 mM KCl was added to 100 μL of 6 IU/mL thrombin solution in normal saline, and the mixture kept at room temperature for 5 min. The citrate-stabilized serum (100 μL) was incubated for 120 s at 37 °C, then the thrombin-aptamer complex solution (100 μL) was added, and the clotting time measured using a coagulation analyzer (MiniLab-701, Unimed, Russia).

### Nuclease cleavage assay

Oligonucleotides were dissolved at a concentration of 50 μM in 30 μL of reaction buffer containing 20 mM KCl. Samples were heated to 100 °C and annealed before the digest. One unit of S1 nuclease (Thermo Scientific) was added to the aptamer samples. After incubation for 10 and 30 min at 25 °C, reaction mixtures were precipitated with 2% lithium perchlorate in acetone and analyzed by electrophoresis. Analysis was performed with a 20% polyacrylamide gel in 7 M urea and 0.1 M TBE at 25 °C.

### Molecular dynamics and docking

TBA-N8 MD simulations were carried out using the Amber 10 program suite^30^. The influence of the solvent was simulated with a TIP3P water model^31^. The simulation was performed under periodical boundary conditions in a rectangular box. The parameters for the inter-atomic energy calculation were taken from the force field parmbsc0^32^. Partial charges on NI atoms were calculated by first individually optimizing the NI residues (a methyl group was used instead of the sugar moiety) using DFT with hybrid exchange-correlation functional B3L LYP (Becke three-parameter (exchange), Lee, Yang and Parr (correlation))^33^,^34^,^35^ and the aug-cc-pVTZ basis sets. Single point calculations were then performed at the MP2/aug-cc-pVTZ level. Finally, for the electron density distribution determined with DFT/B3LYP, partial atomic charges were obtained by charge fitting of the electrostatic potential at points selected according to the Merz-Singh-Kollman scheme^36^. All quantum mechanic simulations were carried out using the Gaussian 09 program^37^. A K^+^cation was placed at the centre of the aptamer between the G-tetrads to stabilize the structure. Adding Na^+^ ions neutralized the negative charges and 1947 water molecules were employed for solvation. The structures were minimized and an MD simulation performed as previously described for the TBA analog in complex with thrombin^38^ and the energies of the aptamers were estimated by using the MM-GBSA approach. The total mechanical energy of the molecule in gas phase (E_MM_) was calculated as the sum of the electrostatic energies (E_eq_), van der Waals energies (E_vdW_), and the energies of internal strain (bonds, angles and dihedrals) by using a molecular-mechanics approach. The free energy of solvation was calculated as the sum of the polar and nonpolar contributions. The polar contribution (E_GB_) was computed using the Generalized Born (GB) method^39^ and the algorithm developed by Onufriev *et al*. for calculating the effective Born radii^40^. The non-polar contribution to the solvation energy (E_surf_), which includes solute-solvent van der Waals interactions and the free energy of cavity formation in solvent, was estimated from a solvent-accessible surface area (SASA).

The docking procedure with a partially flexible aptamer was performed using Autodock 4.2^41^. The quadruplex core of TBA/TBA-N8 was fixed, while in the loops heterocyclic bases could rotate around the glycosidic bonds. Naturally, this only allowed for a partial accounting of the actual conformational diversity in the loops. To consider at least several possible loop arrangements, we used several conformers of each aptamer taken from MD trajectories and differing in loop arrangements (not just 50 ns ones), in our docking experiments. The results shown in Supplementary Figure S10 and Table 2 refer to the conformers that demonstrated the best affinity for thrombin (the highest binding energy).

The protein and the aptamers were prepared for docking by AutodockTools (ADT Version 1.5.4), and the partial atomic charges on the aptamer atoms that were calculated for MD simulations were preserved. The partial atomic charges on protein atoms were calculated using the Gasteiger-Hückel method^42^.

Two types of docking experiments were performed. The first type was ‘rigid’ docking with a fixed protein body. The second type was ‘half-rigid’ docking with flexible side chains of the key amino acid residues. The thrombin model used in the ‘half-rigid’ docking of the aptamers to exosite I and the proximal area was taken from PDB:1HAO (the amino acids with flexible side chains are His71, Arg73, Thr74, Arg75, Tyr 6, Glu77, Arg77A, Asn78, Glu80 and Tyr117). The model of the thrombin/heparin complex used for docking the aptamers to exosite II and the proximal area was taken from PDB:1XMN (the amino acids with flexible side chains are Arg93, Arg101, Arg126, Arg233 and Lys240). Three-dimensional grid maps (126 × 126 × 126 points) for each atom type were computed using AutoGrid4. In the case of ‘rigid’ docking to the whole protein, the grid centre was placed at the geometric centre of the protein, and the size of a lattice cell was 0.5 Å. In the case of docking to exosite I area, the grid centre was placed into the CA atom of Lys70 with a lattice cell of 0.375 Å. In the case of docking to the exosite II area, the lattice cell was also 0.375 Å, and the grid centre was placed into the CD1 atom of Ile103. Electrostatic and desolvation maps of the protein were calculated. The Lamarckian genetic algorithm (GA-LS)^43^, a hybrid of a genetic algorithm and a local search algorithm, was employed for identifying the most probable binding site. The number of GA-LS runs was 50, the maximum number of energy estimations was 2500000, the maximum number of generations was 27000, and the mutation and crossover rates were 0.02 and 0.8, respectively. Pseudo-Solis & Wets parameters were used for the local search, and the number of iterations was 300. Starting positions and aptamer conformations were random. The torsion angle rotation step was 50°. After docking, all the generated structures were clustered up with RMS tolerance of 2 Ǻ from the lowest energy. Binding free energies (ΔG) of the aptamer/protein complexes were calculated according to the formula:


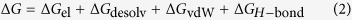


The electrostatic interaction free energy (ΔG_el_) was estimated using a distance-dependent dielectric function of Mehler and Solmajer. The van der Waals interaction free energy (ΔG_vdW_) was estimated using the L-J potential and atomic parameters from the AMBER Force Field. For scoring the H-bond energy ΔG_H-bond_, the 10/12 potential was used with a maximal well depth of 5 kcal/mol at 1.9 Å for hydrogen bonds with oxygen and nitrogen atoms, and then multiplied by the function estimation rate from the ideal H-bonding geometry. For estimating the desolvation energy ΔG_desolv_, the atomic fragmental volume and solvation parameters derived from the method of Stouten *et al*.^44^ were used. Each term was multiplied by a semi-empirical weighting constant by fitting to the binding affinity data of a training set of complexes with known structures. The scoring function in the docking procedure also involved ΔG_tors_, the conformational entropy defined via the conformational mobility of the aptamer and calculated using a number of dihedral angles. In our case, ΔG_tors_ could not be evaluated precisely (the weighting constant for ΔG_tors_ might be incorrect because the regression multipliers for this parameter have been used for chemical structures, which are different from the aptamers we used).

## Additional Information

**How to cite this article**: Tsvetkov, V. B. *et al*. A Universal Base in a Specific Role: Tuning up a Thrombin Aptamer with 5-Nitroindole. *Sci. Rep*. **5**, 16337; doi: 10.1038/srep16337 (2015).

## Supplementary Material

Supplementary Information

## Figures and Tables

**Table 1:** Modified TBA analogs.

ON code	Sequence^a^, 5′-3′	Clotting time^b^, s	T_m_^uv^ (°C)	T_m_^cd^ (°C)
TBA	GGTTGGTGTGGTTGG	27.0	51.9	52.2
TBA-N7	GGTTGG**N**GTGGTTGG	26.2	43.0	43.3
TBA-N8	GGTTGGT**N**TGGTTGG	117.0	51.6	52.1
TBA-N9	GGTTGGTG**N**GGTTGG	22.4	38.5	39.1
TBA-N1e	**N**GGTTGGTGTGGTTGG	26.0	51.1	51.4
TBA-N15e	GGTTGGTGTGGTTGG**N**	27.5	51.2	50.9

^a^**N**—5-nitroindole.

^b^Measured at 37 °С and a thrombin concentration of 6 IU/mL; the aptamer concentration was 0.15 μM. Each clotting time value is the mean of three measurements. The standard deviation does not exceed 10%.

**Table 2:** Estimates of the thrombin-aptamer binding energies.

Aptamer code	Thrombin exosite	Aptamer binding mode	Binding energy, kcal/mol		
			ΔG_el_	ΔG_vdW_ + ΔG_h-bond_ + ΔG_desolv_	ΔG^a^
TBA-N8	I	Via TT-loops	−2.24	−7.07	−9.32
		Via the central loop	−2.64	−6.65	−9.29
	II	Via TT-loops	−2.29	−8.25	−10.54
		Via the central loop	−3.66	−7.9	−11.56
TBA	I	Via TT-loops	−2.55	−6.26	−8.81
		Via the central loop	−3.26	−4.11	−7.37
	II	Via TT-loops	−3.03	−7.63	−10.66
		Via the central loop	−3.65	−6.50	−10.15

^a^For details of ΔG calculations, see the experimental section.
