# Supplementary material for: A Universal Base in a Specific Role: Tuning up a Thrombin Aptamer with 5-Nitroindole
Source: Sci Rep. 2015 Nov 17;5:16337. doi: 10.1038/srep16337 (PMC4648099; doi:10.1038/srep16337)
Supplement: Supplementary Information [file srep16337-s1.pdf]

Supplementary data

A UNIVERSAL BASE IN A SPECIFIC ROLE: TUNING UP A THROMBIN APTAMER WITH 5-NITROINDOLE

Vladimir B. Tsvetkov,[2,3] Anna M. Varizhuk,[1,2] Galina E. Pozmogova,[2] Igor P. Smirnov,[2] Natalia A. Kolganova[1] and Edward N. Timofeev[1*]

[1] W. A. Engelhardt Institute of Molecular Biology Russian Academy of Sciences, Moscow 119991, Russia

[2] Institute for Physical-Chemical Medicine, Moscow 119435, Russia

[3] Topchiev Institute of Petrochemical Synthesis Russian Academy of Sciences, Moscow 119991, Russia

V. B. T. and A. M. V. contributed equally to this work.

*To whom correspondence should be addressed. Tel: +7 499 135 6591; Fax: +7 499 135 1405; Email: edward@eimb.ru

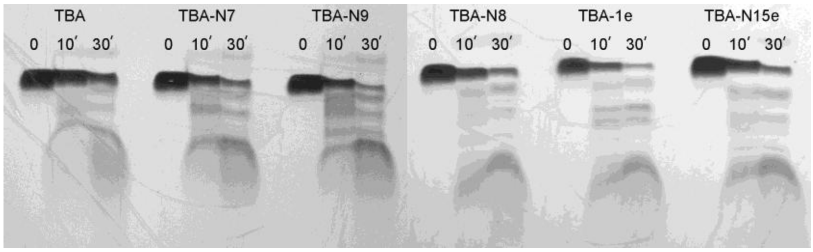

Figure S1. Hydrolysis of TBA and the modified aptamers by S1 nuclease at 37°C in reaction buffer containing 20 mM KCl.

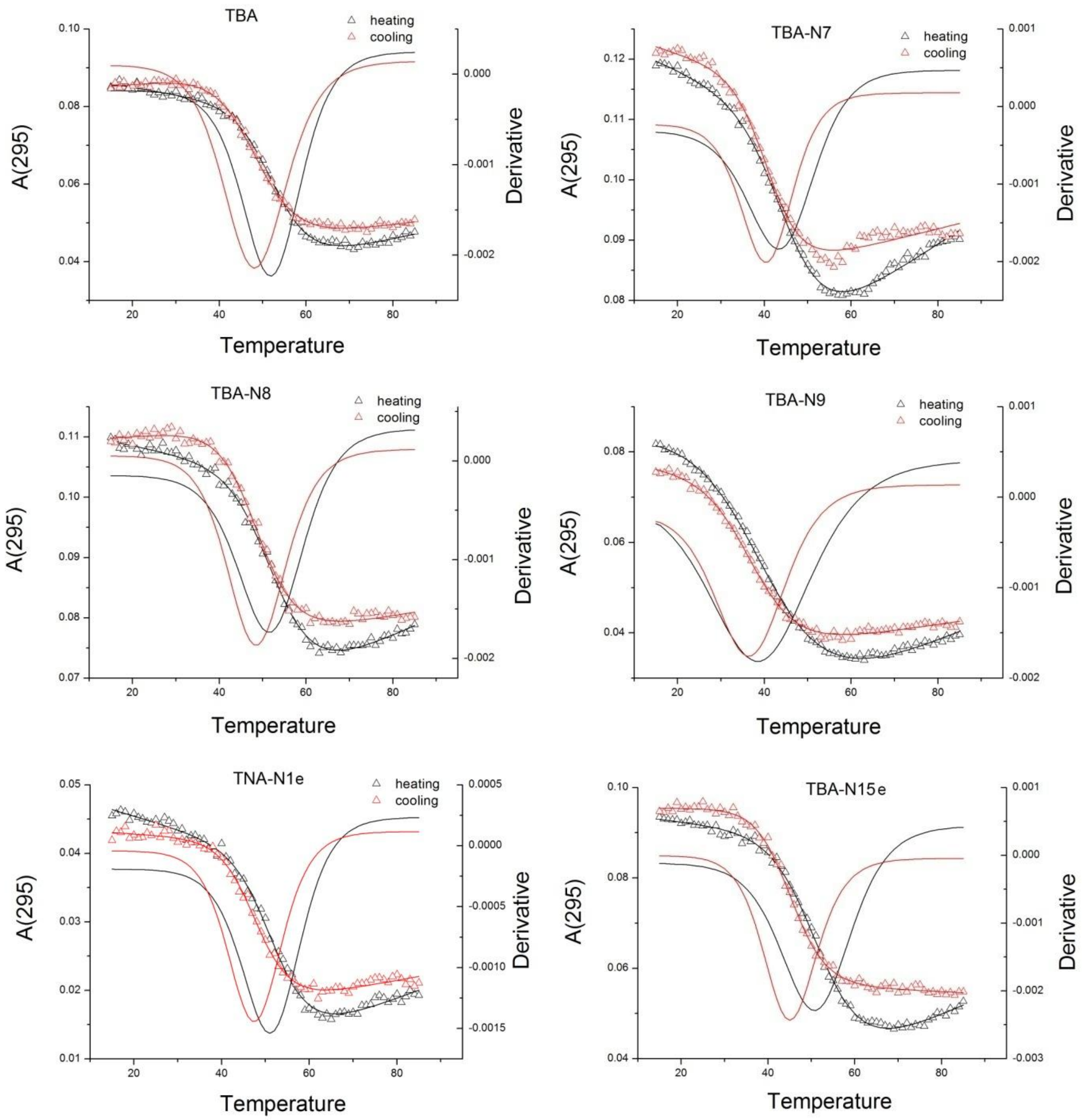

Figure S2. UV-melting profiles and their first derivatives for modified aptamers and TBA. Melting experiments were carried out at 295 nm in 10 mM sodium cacodylate (pH 7.2) and 100 mM KCl. The oligonucleotide concentration was in the range 2-6 μM.

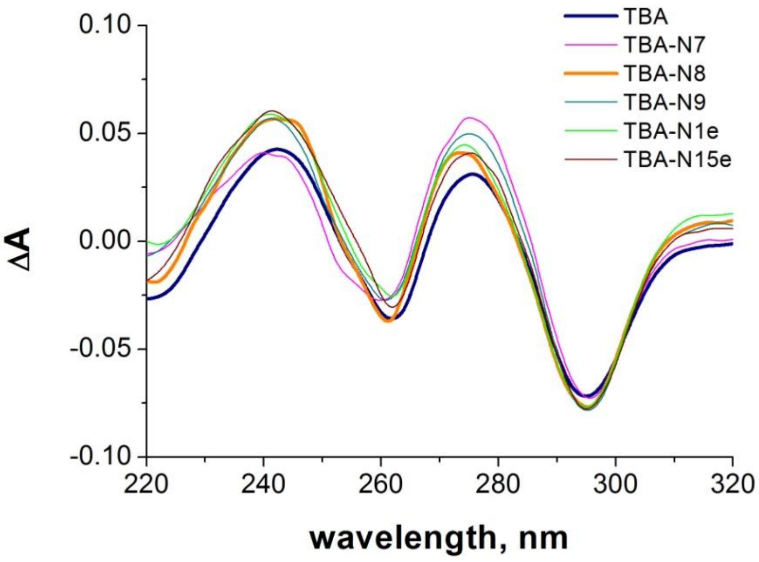

Figure S3. Thermal difference spectra (80°C vs. 20°C) of TBA and modified aptamers at concentration of 5 μM in 10 mM sodium cacodylate (pH 7.2) and 100 mM KCl.

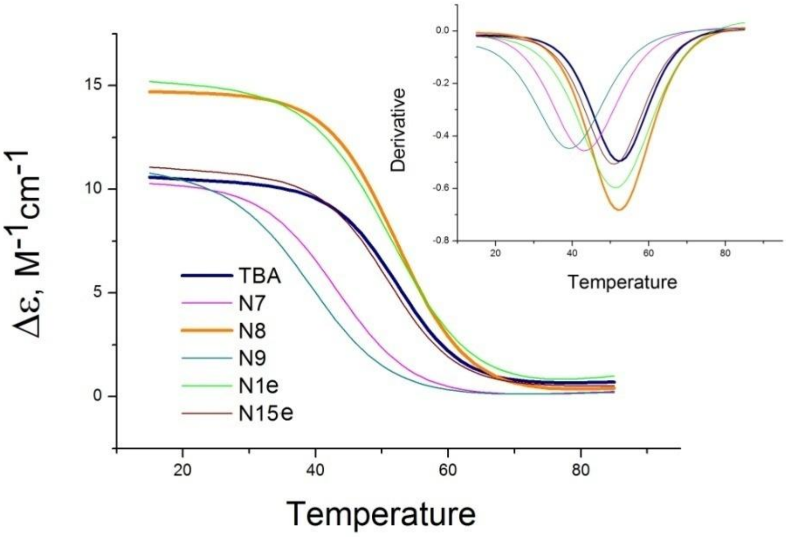

Figure S4. CD melting profiles and their first derivatives for modified aptamers and TBA. Melting experiments were carried out at 295 nm in 10 mM sodium cacodylate (pH 7.2) and 100 mM KCl. The oligonucleotide concentration was 5 μM.

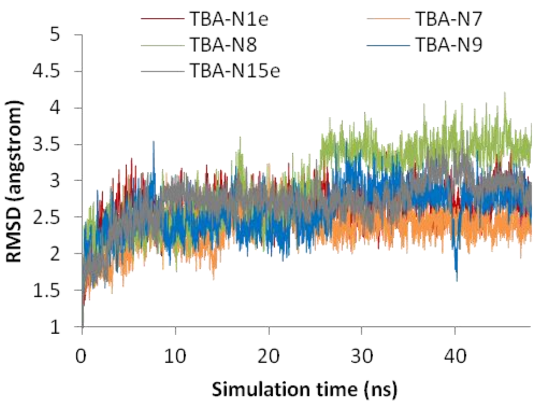

Figure S5. MD simulation results for TBA analogs. The RMSD profiles roughly plateau after ≈40 ns, conformational fluctuations are minimal.

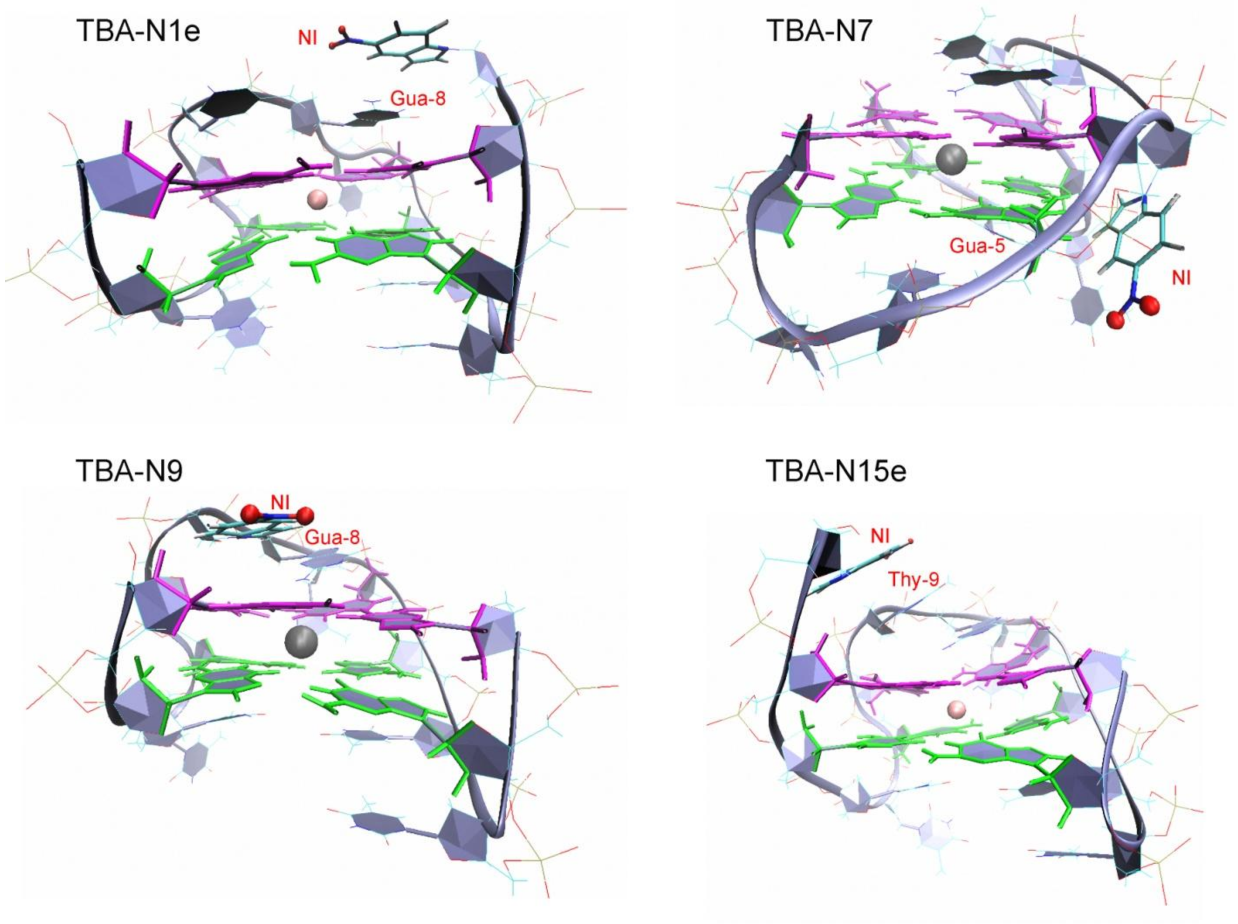

Figure S6. Stable aptamer conformations (50 ns snapshots).

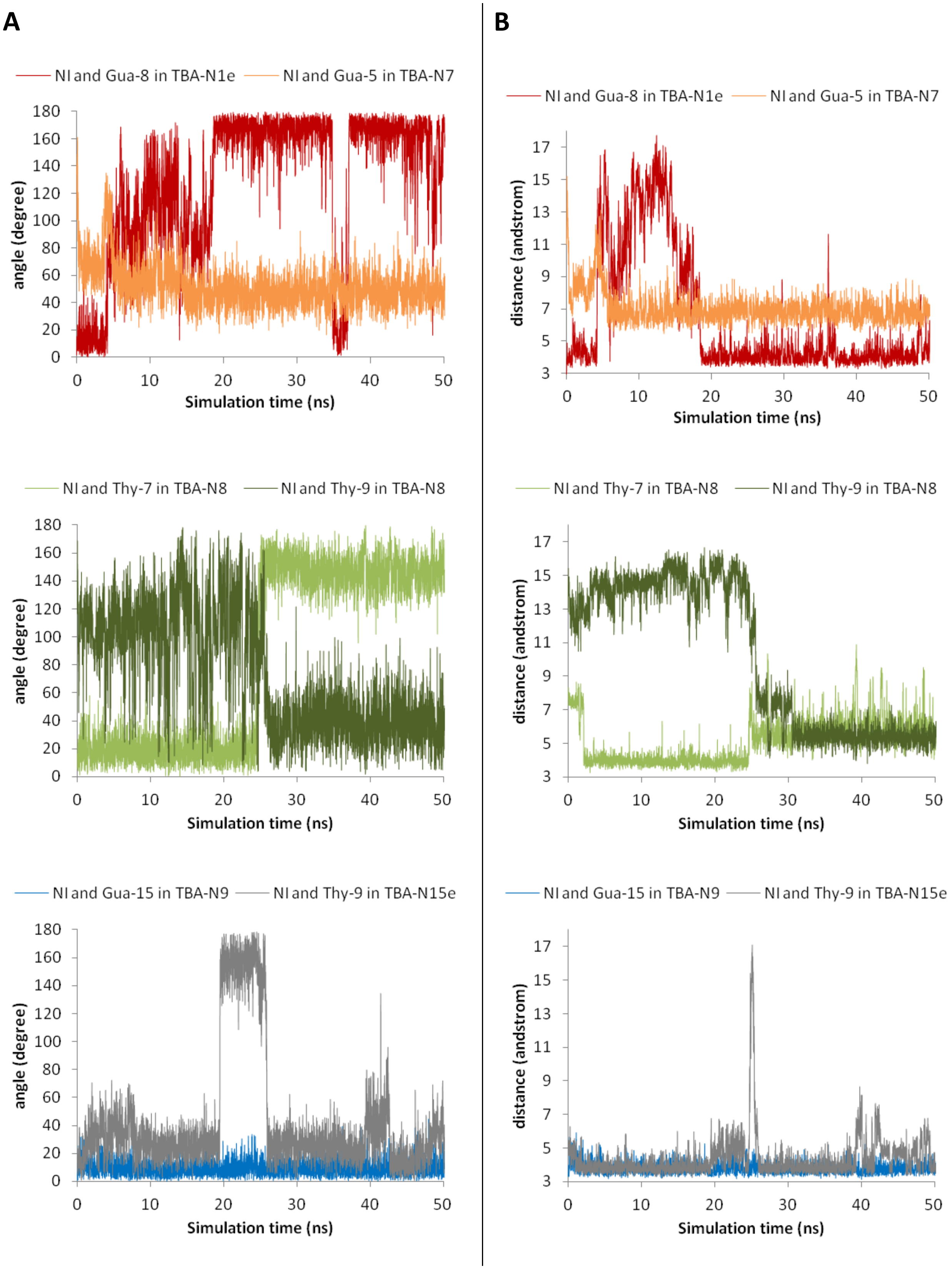

Figure S7. Spatial orientation of the NI residue in TBA analogs (A) Angles between normals to NI planes and the proximal nucleobases. (B) Distances between COMs (centre of mass) of NI and the proximal nucleobases.

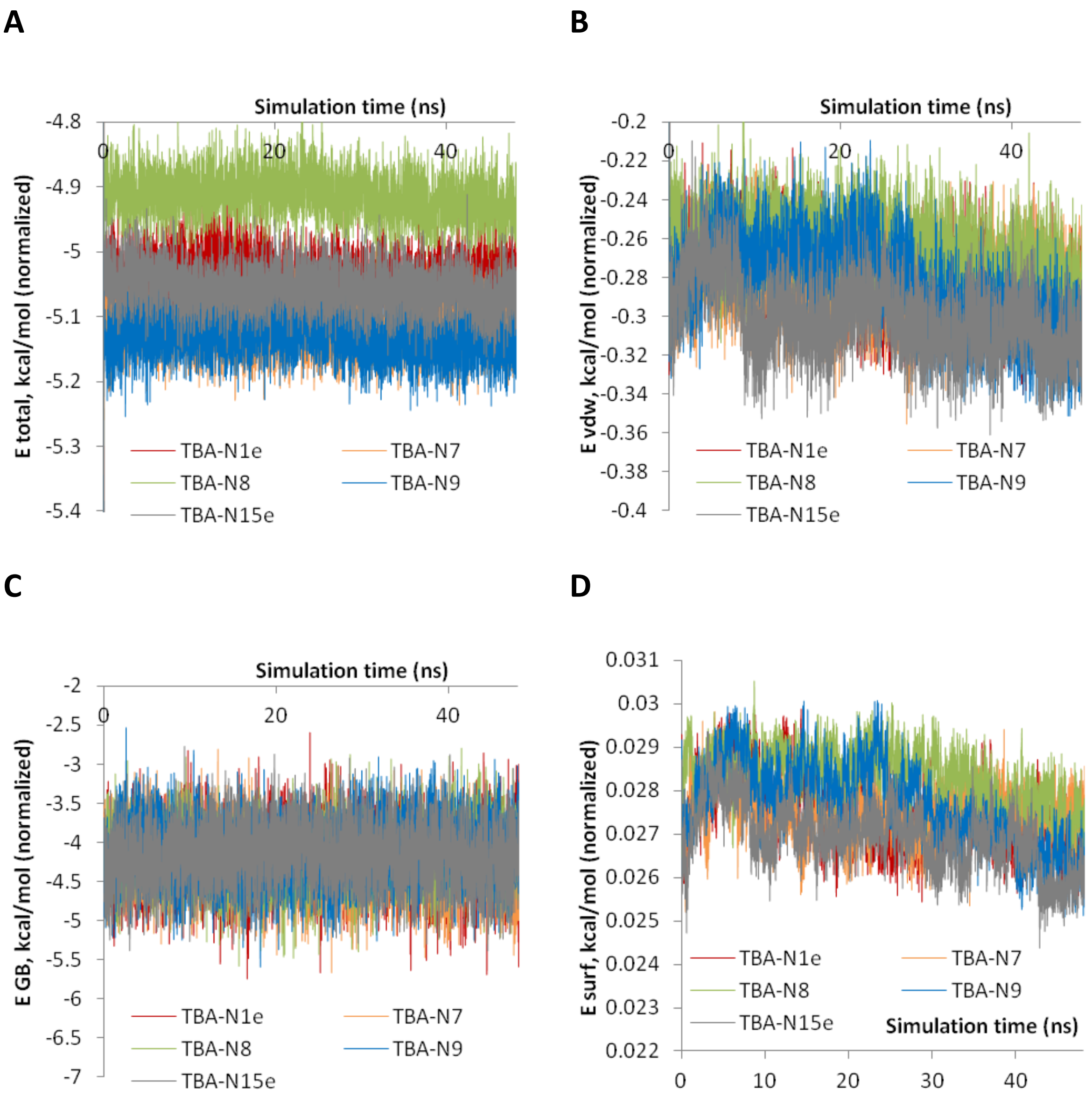

Figure S8. Energy profiles for TBA analogs. (A) Total potential energy ($E_{total} = E_{MM} + E_{solv}$). (B) Van der Waals energy. (C) Solvation energy, the polar contribution. (D) Solvation energy, the non-polar contribution. The energies were normalized by the number of atoms in an aptamer.

A B

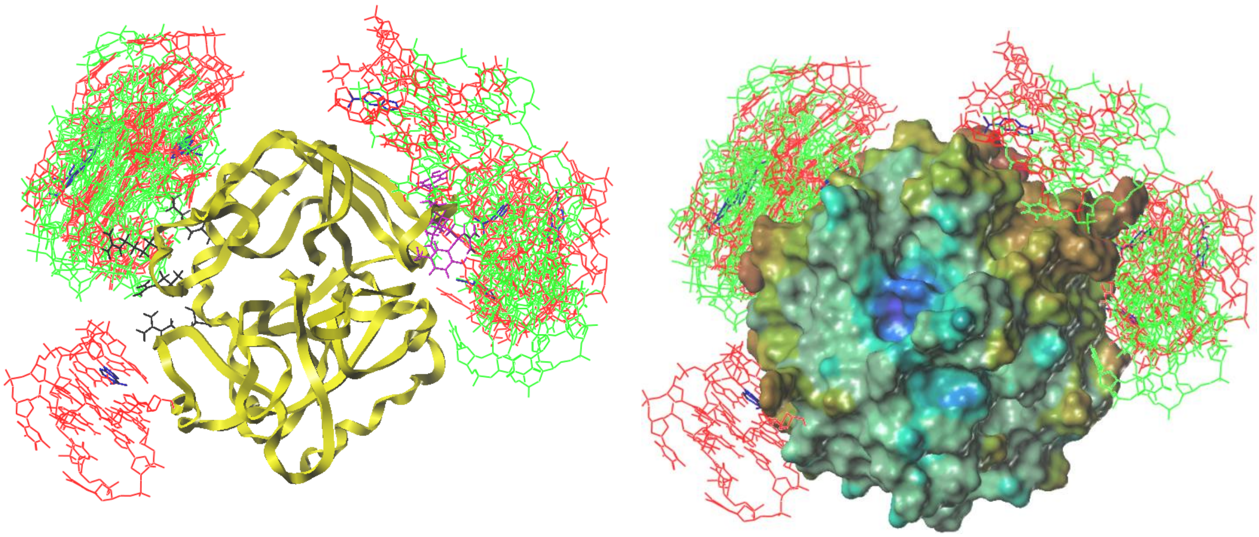

Figure S9. ‘Rigid’ docking results. Putative binding sites of TBA-N8 (red) and unmodified TBA (green). Only the most energetically favorable conformations of each TBA-N8 and TBA conformer cluster are shown. (A) Thrombin major body is yellow, Arg75,Tyr76,Glu77,Arg77A and Ty117 side residues in thrombin exosite I are pink; Arg93, Arg101, Arg233 and Lys240 side residues in thrombin exosite II are black. (B) The protein surface is colored according to the electrostatic potential: blue (greenish) indicates negative charge; red indicates positive charge.

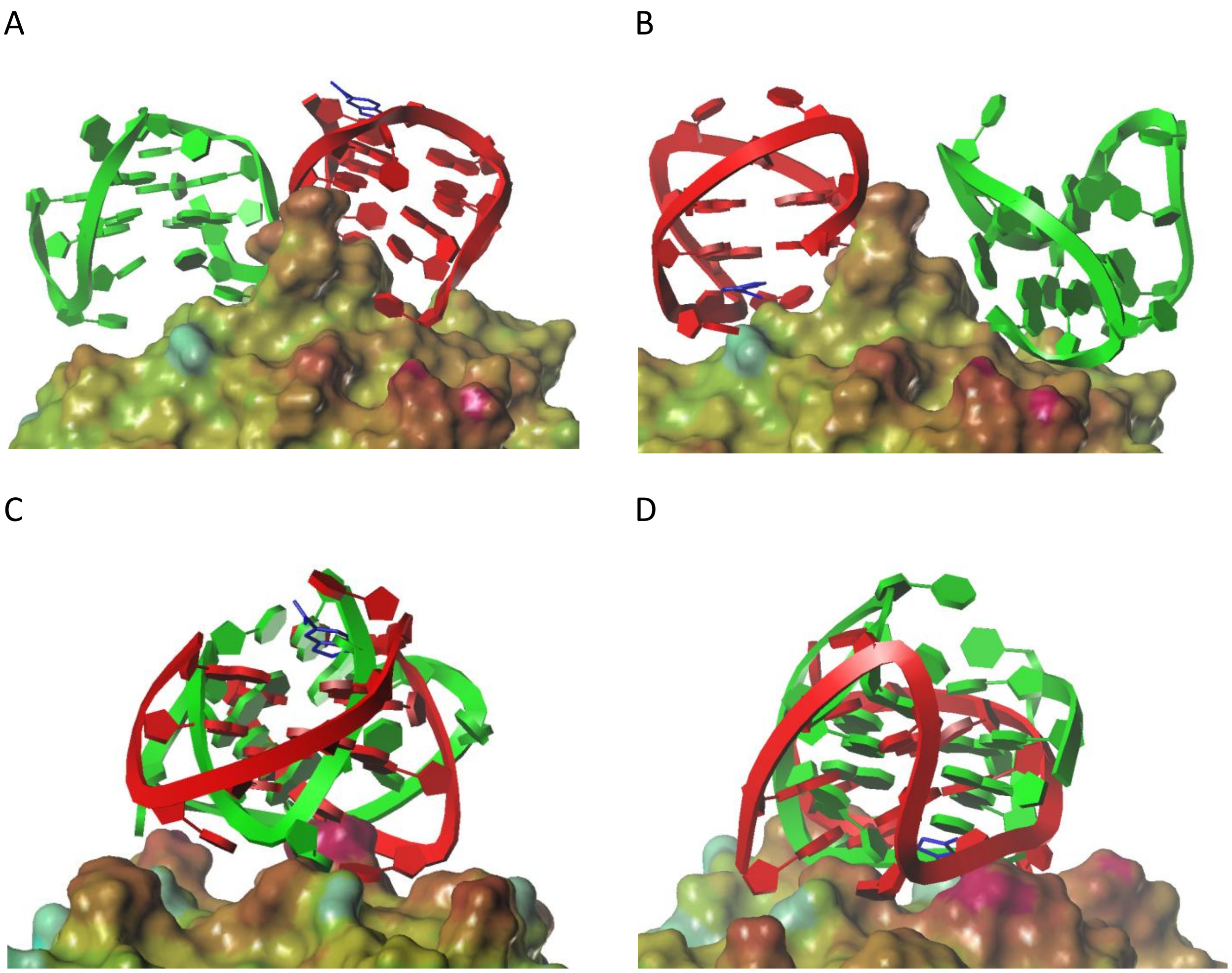

Figure S10. Results of docking TBA and TBA-N8 to thrombin exosites I and II. Only the conformers corresponding to the best binding energies are shown for each site and each interaction mode. The protein surface is colored according to the electrostatic potential: blue (greenish) indicates negative charge; red indicates positive charge. TBA is green, TBA-N8 is red. The NI residue is blue. (A) and (B) Aptamers in thrombin exosite I, binding via TT loops and via the central loop, respectively. (C) and (D) Aptamers in thrombin exosite II, binding via TT loops and via the central loop, respectively.

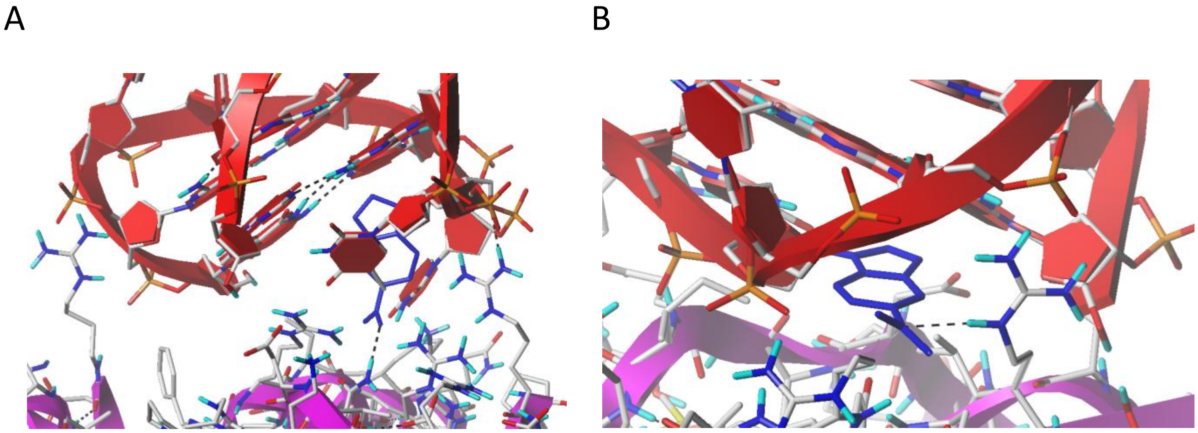

Figure S11. Hydrogen bond interactions of NI residue. (A) Asn 179 in exosite II. (B) Arg 75 in exosite I.

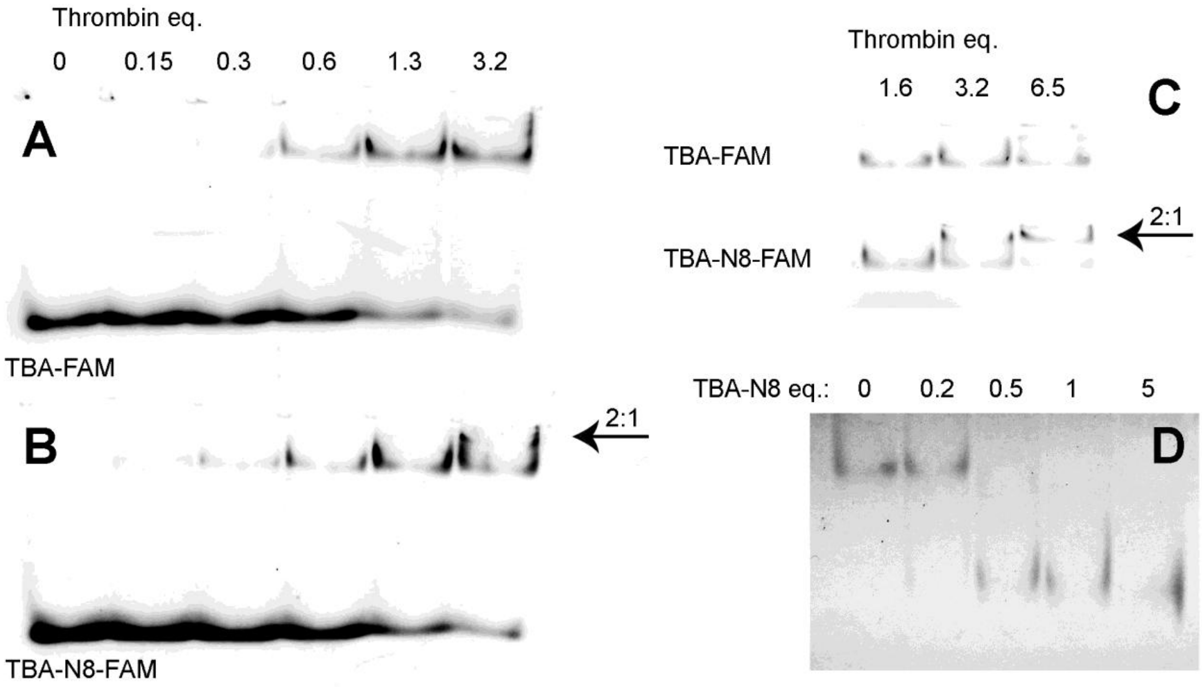

Figure S12. Native gel-electrophoresis. Binding of TBA-FAM (A,C) and TBA-N8-FAM (B,C) at a concentration of 5 μM at 20°C with increasing concentrations of thrombin. Arrow points to the 2:1 thrombin – (TBA-N8-FAM) complex. Binding of thrombin (4 μM) at 20°C with increasing concentrations of TBA-N8 (D).

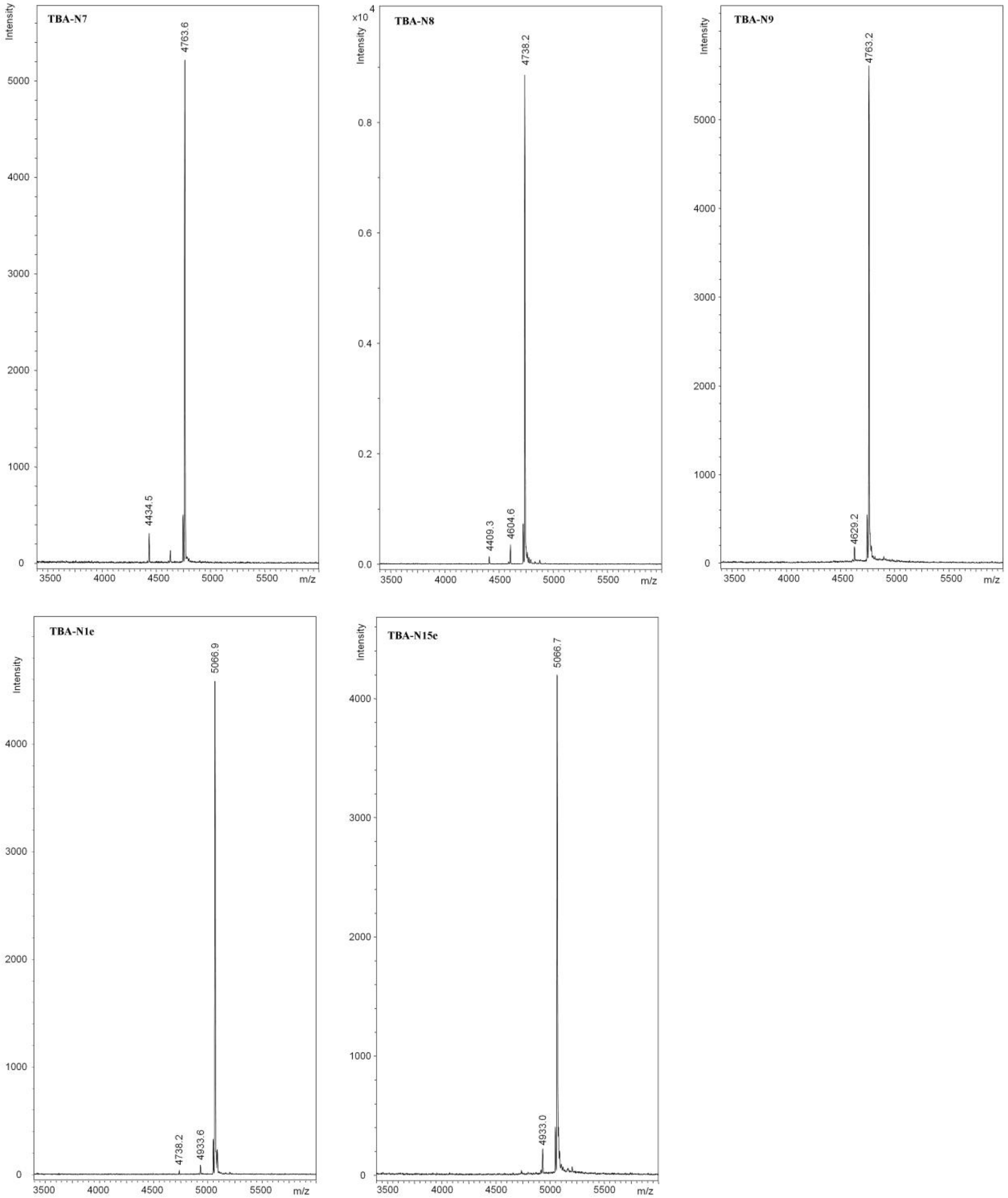

Figure S13. MALDI mass spectra of modified oligonucleotides.

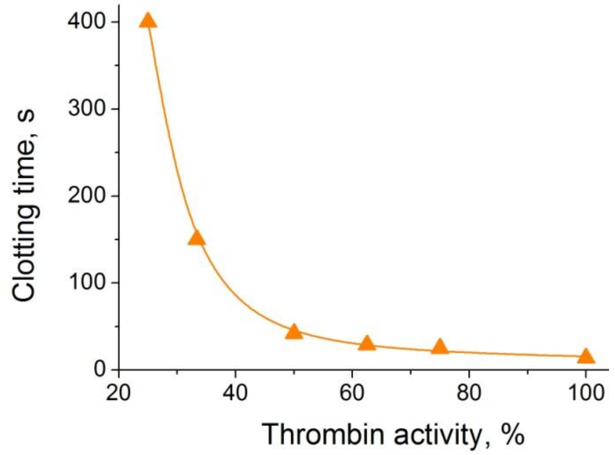

Figure S14. Calibration curve for converting clotting time values into thrombin activity. A series of thrombin dilutions was prepared and mixed with blood plasma. Clotting times were measured. Each clotting time value is an average of three measurements.

Table S1. Estimates of the thrombin-aptamer binding energies.

Apt. code	Thr. exosite	Aptamer binding mode	Binding energy, kcal/mol				ΔU^{b}, kcal/mol	ΔU_{ligand}, kcal/mol
			ΔG_{el}	ΔG_{vdW} + $\Delta G_{h\text{-}bond}$ + ΔG_{desolv}	ΔG	ΔG_{tors}^{a}		
TBA	I	Via TT-loops	-2.55	-6.26	-8.81	28.94	1.91	12.69
		Via the central loop	-3.26	-4.11	-7.37		1.46	12.33
	II	Via TT-loops	-3.03	-7.63	-10.66		8.69	11.74
		Via the central loop	-3.65	-6.50	-10.15		7.71	11.71
TBA-N8	I	Via TT-loops	-2.24	-7.07	-9.32	28.64	-8.76	0.88
		Via the central loop	-2.64	-6.65	-9.29		-10.25	0.16
	II	Via TT-loops	-2.29	-8.25	-10.54		0.27	4.04
		Via the central loop	-3.66	-7.9	-11.56		0.16	2.91
TBA-N1e	I	Via TT-loops	-2.28	-6.15	-8.43	31.02	-6.60	5.54
		Via the central loop	-2.95	-5.5	-8.47		-4.85	6.01
	II	Via TT-loops	-1.20	-7.20	-8.41		0.30	3.94
		Via the central loop	-1.39	-8.23	-9.62		0.07	7.02
TBA-N15e	I	Via TT-loops	-1.72	-8.73	-10.44	31.02	-13.53	1.01
		Via the central loop	-2.87	-7.07	-9.94		0.92	3.03
	II	Via TT-loops	-1.81	-8.33	-10.14		-3.68	1.14
		Via the central loop	-3.66	-5.89	-9.56		-1.17	4.42
TBA-N7	I	Via TT-loops	-3.52	-6.70	-10.22	29.23	-9.28	2.29
		Via the central loop	-2.21	-4.88	-7.09		-9.81	1.36
	II	Via TT-loops	-1.68	-9.38	-11.07		2.99	3.21
		Via the central loop	-2.02	-5.68	-7.70		-2.66	2.24
TBA-N9	I	Via TT-loops	-3.26	-6.35	-9.61	29.23	-5.72	4.36
		Via the central loop	-3.79	-6.37	-10.15		-3.68	6.00
	II	Via TT-loops	-2.89	-8.92	-11.81		-0.81	2.12
		Via the central loop	-3.18	-5.83	-9.02		-0.65	4.75

[a]This term was excluded from ΔG because its absolute values are hard to estimate accurately, however, it provides a general idea of the conformational entropy contribution.

[b]This term (the internal strain) cannot be compared directly for exosites 1 and 2 because of the conditions of our ‘half-rigid’ docking procedure.
